# Role of cancer stem cell markers ALDH1, BCL11B, BMI-1, and CD44 in the prognosis of advanced HNSCC

**DOI:** 10.1007/s00066-020-01653-5

**Published:** 2020-06-25

**Authors:** Mark Jakob, Kariem Sharaf, Markus Schirmer, Martin Leu, Stefan Küffer, Mattis Bertlich, Friedrich Ihler, Frank Haubner, Martin Canis, Julia Kitz

**Affiliations:** 1 Department of Otolaryngology, University Hospital, LMU Munich, Marchioninistraße 15, 81377 Munich, Germany; 2 Department of Otolaryngology, University Medical Center Göttingen, Göttingen, Germany; 3 Department of Radiation Oncology, University Medical Center Göttingen, Göttingen, Germany; 4 Department of Pathology, University Medical Center Göttingen, Göttingen, Germany; 5 German Center of Vertigo and Dizziness, University Hospital, LMU Munich, Munich, Germany

**Keywords:** Head and neck cancer, Survival, Primary radiochemotherapy, Surgical treatment, Molecular markers

## Abstract

**Purpose:**

Cancer stem cells (CSCs) are held accountable for the progress of head and neck squamous cell carcinoma (HNSCC). In the presented study, the authors evaluated the prognostic value of CSC markers in two particular HNSCC cohorts.

**Methods:**

This two cohort study consisted of 85 patients with advanced stage HNSCC, treated with primary radio(chemo)therapy (pRCT), and 95 patients with HNSCC, treated with surgery and partially adjuvant radio(chemo)therapy. Overall survival (OS), disease-free survival (DFS), and disease-specific survival (DSS) were assessed. Samples were assessed for the expression of different molecular stem cell markers (ALDH1, BCL11B, BMI‑1, and CD44).

**Results:**

In the pRCT cohort, none of the baseline patient and tumor features exhibited a statistically significant relation with survival in either the cohort or the human papillomavirus (HPV)-stratified subcohorts. High expression of BMI‑1 significantly decreased OS and DFS, while high expression of CD44 decreased all modes of survival. Multivariate analysis showed significant prognostic influence for all tested CSC markers, with high BMI‑1 and CD44 decreasing survival (BMI-1: OS, DFS, DSS; CD44: OS, DFS) and high ALDH1 and BCL11B showing a beneficial effect on survival (ALDH1: OS, DFS; BCL11B: OS, DSS). In the surgical cohort, classical prognosticators such as HPV status, R1 resection, and nodal status in HPV-negative HNSCC played a significant role, but the tested CSC markers showed no significant effect on prognosis.

**Conclusion:**

Although validation in independent cohorts is still needed, testing for CSC markers in patients with advanced or late stage HNSCC might be beneficial, especially if many comorbidities exist or disease is irresectable. The findings might guide the development and earlier use of targeted therapies in the future.

**Electronic supplementary material:**

The online version of this article (10.1007/s00066-020-01653-5) contains supplementary material, which is available to authorized users.

## Introduction

Head and neck squamous cell cancer (HNSCC) is one of the most prevalent cancers worldwide [1]. Risk factors include tobacco and betel nut use, alcohol consumption, as well as combined abuse of tobacco and alcohol, frequent mouthwash use, and exposure to human papillomavirus (HPV) [2]. Of the mentioned risk factors, HPV exposure seems to cause an increasing fraction of HNSCC incidence worldwide, including Germany [3, 4].

Clinical outcome is dependent on tumor size, locoregional spread, resection margins, extracapsular extension, lymphovascular invasion, and distant metastasis [5, 6]. Alongside TNM classification, HPV status can serve as a marker to predict survival probabilities, which eventually led to the development of a new TNM classification system for p16-positive oropharyngeal carcinomas [6–8].

Despite improvements in diagnosis and treatment of HNSCC over the past decades, long-term survival has improved only marginally [5, 9, 10]. To improve survival in HNSCC patients, investigations into the underlying molecular and phenotypic changes associated with tumorigenesis, disease progression, and metastasis are necessary.

More recent studies have shown that survival of cancer stem cells (CSCs; also known as tumor-initiating cells) may contribute to tumor progression, metastasis, and recurrence of HNSCC [11–13]. Moreover, CSCs may contribute to conventional therapy resistance and are responsible for tumor progression and recurrence [13].

BMI‑1, ALDH1, and CD44 are amongst the most common and most studied CSC markers in HNSCC [14]. BMI‑1 (B-lymphoma Moloney murine leukemia virus insertion region-1) acts in the self-renewal ability of stem cells. High expression of BMI‑1 in cancer was related to epithelial–mesenchymal transition (EMT) and poor prognosis [14, 15]. ALDH1 (aldehyde dehydrogenase 1) is important for the maintenance and differentiation of stem cells [16]. High ALDH1 expression was associated with advanced tumor stage, tumor size, and lymph node metastasis [17]. CD44 is a surface glycoprotein and a common CSC marker in several human tumor entities [18]. In oral cancer, high expression CD44 was linked to adverse outcome [19]. In strong co-expression to BMI‑1, BCL11B (B-cell lymphoma/leukemia 11B) was identified as an additional CSC marker in HNSCC that is otherwise observed in both embryogenesis and tumor suppression [14, 20].

While there is already some data on the correlation with disease characteristics such as tumor size, lymph node metastasis, and grading, the correlation of CSC marker expression and prognosis of survival is not well studied. In this study, the authors therefore tested the prognostic value of CSC markers in distinct patient cohorts with mostly advanced HNSCC.

## Material and methods

### Patients

In a single-center retrospective two cohort study, 184 patients were included. One cohort (primary radio[chemo]therapy cohort, pRCT cohort) included 89 patients. Continuous cases were included that were (1) diagnosed with histopathologically confirmed advanced stage head and neck squamous cell carcinoma between November 1998 and May 2012, and (2) treated with primary radiotherapy or pRCT. All diagnostic and treatment procedures (3) had to be performed in the same tertiary referral center. Patients that (1) received pRCT in palliative intent with reduced planned radiation dose and with (2) missing clinical data or (3) retracted informed consent were excluded. Further important reasons for exclusion included (4) insufficient amounts of tumor tissue, (5) damaged specimen or failure of immunohistochemistry control stainings.

The other cohort (surgical cohort) included 95 patients that were (1) diagnosed with head and neck squamous cell carcinoma between August 2001 and June 2015 and (2) treated with surgical resection. Of these patients, 72 also underwent adjuvant radiotherapy or radiochemotherapy. All diagnostic and treatment procedures (3) had to be performed in the same tertiary referral center. Many patients needed to be excluded due to (1) missing informed consent. Also patients with (2) relapse at diagnosis, (3) distant spread at diagnosis, or (4) previous radiation therapy for any cause before surgical treatment, (5) missing clinical data, as well as (6) insufficient amounts of tumor tissue, (7) damaged specimen or failure of immunohistochemistry control stainings were excluded.

Clinical data, including risk factors such as smoking and drinking habits, as well as data regarding disease stage and therapy, were obtained from the medical documentation of the patients. Histologic samples were collected and analyzed as described below.

In the pRCT cohort, out of 89 patients in total, HPV testing showed valid results in 88 patients and molecular testing of all of the tested markers could be obtained in the histologic samples of 85 patients. Detailed patient characteristics of these 85 patients are described in Table 1. In the surgical cohort, HPV and molecular testing could be obtained in all 95 patients. Patient characteristics of the 95 patients of the surgical cohort are described in Table 2. All data is displayed for the entire sample set regardless of the HPV status and separately for the HPV-distinct subcohorts.

**Table 1:** General patient characteristics, risk factors, and disease stage of the primary radio(chemo)therapy (pRCT) cohort. All continuous data variables are displayed as mean ± standard deviation

Characteristics *pR(C)T cohort*		Cohort (*n* = 85)	HPV-neg. subcohort (*n* = 53)	HPV-pos. subcohort (*n* = 31)
*Gender*	Male	82.4%	81.1%	83.9%
	Female	17.6%	18.9%	16.1%
*Age at diagnosis (years)*		58.3 ± 11.2	55.6 ± 10.7	62.4 ± 10.7
*Relapse at diagnosis*	Positive	14.1%	9.4%	22.6%
*Substance abuse*	Negative	14.1%	11.3%	16.1%
	Nicotine (>10 py)	21.2%	18.9%	25.8%
	Alcohol	3.5%	3.8%	3.2%
	Combined (Nicotine + Alcohol)	57.6%	62.3%	51.6%
	Insufficient data	3.5%	3.8%	3.2%
*HPV status*	Positive	36.5%	0%	100%
	Negative	62.4%	100%	0%
	Not determinable	1.2%	0%	0%
*Localization*	Oropharyngeal	35.3%	41.5%	25.8%
	Oral cavity	32.9%	32.1%	35.5%
	Hypopharyngeal	11.8%	9.4%	12.9%
	Laryngeal	7.1%	9.4%	3.2%
	Multi-site	12.9%^a^	7.5%	22.6%
*Staging*	cT 1	4.7%	5.7%	3.2%
	2	5.9%	1.9%	12.9%
	3	11.8%	11.3%	12.9%
	4	77.6%	81.1%	71.0%
	cN positive	84.7%	88.7%	77.4%
	cN 1	9.4%	11.3%	6.5%
	2	68.2%	71.7%	64.5%
	2b	12.9%	20.8%	16.1%
	2c	24.7%	39.6%	32.3%
	3	7.1%	5.7%	6.5%
	cM1	8.2%^b^	5.7%	9.7%
*Stage (UICC, 7th edition)*	II	3.5%	0%	9.7%
	III	5.9%	5.7%	6.5%
	IV	90.6%	94.3%	83.9%
*Simultaneous malignancy*	Positive	1.2%^c^	1.9%	0%
	Questionable	1.2%	1.9%	0%
*Histology*	G1	3.5%	1.9%	6.5%
	2	76.5%	79.2%	71.0%
	3	20.0%	18.9%	22.6%
*Treatment*	Radiotherapy	100%	100%	100%
	3D LatOP	52.9%	62.3%	38.7%
	iMRT	20.0%	17.0%	25.8%
	RA	3.5%	1.9%	6.5%
	Kreuzfeuer	23.5%	18.9%	29.0%
	Calculated dose 66 Gy	9.4%	5.7%	12.9%
	70 Gy	90.6%	94.3%	87.1%
	Paused	18.8%	9.4%	35.5%
	Insufficient data	1.2%	–	3.2%
	Therapy abortion	9.4%^d^	1.9%	22.6%
	Combined chemotherapy	80.0%	81.1%	83.9%
	Platin +	35.3%	24.5%	51.6%
	Platin −	44.7%	52.8%	32.3%
	Paused	18.8%	13.2%	29.0%
	Surgical debulking	1.2%	0%	3.2%
*Status after pR(C)T*	Complete remission	54.1%	56.6%	51.6%
	Partial remission	23.5%	26.4%	19.4%
	Stable disease	7.1%	3.8%	12.9%
	Progressive disease	7.1%	7.5%	3.2%
	Insufficient data	8.2%	5.7%	12.9%
*Relapse after pR(C)T*	Relapse in total	35.3%	39.6%	25.8%
	Locoregional relapse	34.1%	39.6%	22.6%
*Follow-up time (months)*	Median	15.3	14.3	17.8
	Minimum	1.8^d^	1.8	2.6
	Maximum	155.7	155.7	128.2

*pR(C)T* primary radio- or radiochemotherapy, *UICC* Union for International Cancer Control, *3D LatOP* three-dimensional conformal radiation therapy, *iMRT* intensity-modulated radiotherapy, *RA* radiofrequency ablation

^a^Oro- and hypopharyngeal (*n* = 7); hypopharyngeal and laryngeal (*n* = 2); epi-, oro- and hypopharyngeal (*n* = 1), epi- and oropharyngeal (*n* = 1); thereof HPV neg.: oro- and hypopharyngeal (*n* = 2); hypopharyngeal and laryngeal (*n* = 1); epi- and oropharyngeal (*n* = 1)

^b^Pulmonal (*n* = 5), pleural (*n* = 1), intraorbital (*n* = 1), osseous (*n* = 1), cutaneous (*n* = 1); thereof HPV neg.: pulmonal (*n* = 2), pleural (*n* = 1)

^c^Esophageal squamous cell carcinoma (*n* = 1; HPV neg.)

^d^Patient died during therapy

**Table 2:** General patient characteristics, risk factors, and disease stage of the surgical cohort. All continuous data variables are displayed as mean ± standard deviation

Characteristics *Surgical cohort*		Cohort (*n* = 95)	HPV-neg. subcohort (*n* = 67)	HPV-pos. subcohort (*n* = 28)
*Gender*	Male	77.9%	76.1%	82.1%
	Female	22.1%	23.9%	17.9%
*Age at diagnosis (years)*		59.5 ± 10.4	59.8 ± 11.2	58.6 ± 8.6
*Relapse at diagnosis*	Positive	0%	0%	0%
*Substance abuse*	Negative	37.9%	35.8%	42.9%
	Nicotine (>10 py)	28.4%	25.4%	35.7%
	Alcohol	3.2%	4.5%	0%
	Combined (nicotine + alcohol)	30.5%	34.3%	21.4%
	Insufficient data	0%	0%	0%
*HPV status*	Positive	70.5%	0%	100%
	Negative	29.5%	100%	0%
	Not determinable	0%	0%	0%
*Localization*	Oropharyngeal	69.5%	61.2%	89.3%
	Oral cavity	22.1%	28.4%	7.1%
	Hypopharyngeal	5.3%	7.5%	0%
	Laryngeal	3.2%	3.0%	3.6%
	Multi-site	0%	0%	0%
*Staging*	pT 1	5.3%	4.5%	7.1%
	2	23.2%	22.4%	25.0%
	3	41.1%	37.3%	50.0%
	4	30.5%	35.8%	17.9%
	pN positive	72.6%	67.2%	85.7%
	c/pN 0	27.4%	32.8%	14.3%
	1	23.2%	23.9%	21.4%
	2	47.4%	43.3%	57.1%
	2b	34.7%	32.8%	39.3%
	2c	8.4%	7.5%	10.7%
	3	2.1%	0%	7.1%
	ECE positive	17.9%	14.9%	35.7%
	cM1	0%	0%	0%
	R0	95.8%	94.0%	100%
	1	4.2%	6.0%	0%
	2	0%	0%	0%
*Stage (UICC, 7th edition)*	I	1.1%	1.5%	0%
	II	6.3%	9.0%	0%
	III	29.5%	29.9%	28.6%
	IV	63.2%	59.7%	71.4%
*Simultaneous malignancy*	Positive	0%	0%	0%
	Questionable	0%	0%	0%
*Histology*	G1	1.1%	0%	3.6%
	2	81.1%	88.1%	64.3%
	3	17.9%	11.9%	32.1%
*Indication for adjuvant therapy*	adjR(C)T indicated	88.4%	86.6%	92.9%
	adjR(C)T not indicated	7.4%	9.0%	3.6%
	Facultative	4.2%	4.5%	3.6%
	adjR(C)T refused	16.8%	17.9%	14.3%
	Relapse leading to adjR(C)T	3.2%	4.5%	0%
*Treatment*	Radiotherapy	75.8%	73.1%	82.1%
	Calculated dose 60 Gy	5.3%	6.0%	3.6%
	62.4 Gy	34.7%	32.8%	39.3%
	64 Gy	35.8%	34.3%	39.3%
	Paused	37.9%	34.3%	46.4%
	Therapy abortion	2.1%^a^	3.0%	0%
	Combined chemotherapy	60.0%	56.7%	67.9%
	Paused	18.9%	7.5%	17.9%
*Relapse after pR(C)T*	Relapse in total	33.7%	44.8%	7.1%
	Locoregional relapse	26.3%	34.3%	7.1%
*Follow-up time (months)*	Median	36.6	39.6	31.2
	Minimum	4.7	4.8	4.7
	Maximum	162.7	131.0	162.7

*adjR(C)T* adjuvant radio- or radiochemotherapy, *UICC* Union for International Cancer Control

^a^Abortion at 54 Gy of calculated 64 and 58.24 Gy of calculated 62.4 Gy

All clinical samples were obtained with written informed consent during routine surgery or biopsy based on the approval by the ethics committee of the local medical faculty and in compliance with the World Medical Association Declaration of Helsinki.

### Histological samples and immune staining

Specimens were formalin-fixed and paraffin-embedded. Antigen expression was assessed in tissue microarrays (TMAs) of biopsies from primary tumor specimens and represented the average of two to four punches of 1.5 mm diameter, taken randomly from different regions within each specimen. P16 status as a surrogate marker of HPV infection will be referred to as HPV status in the following (p16-specific antibodies: cs56330, Santa Cruz Biotechnology) [21, 22]. Immunohistochemistry (IHC) was performed using ALDH1-, BCL11B-, BMI-1-, and CD44-specific antibodies (ALDH1: ab52492, abcam; BCL11B: HPA049117, Sigma Aldrich; BMI-1: #6964, Cell Signaling Technology; CD44: #3570, Cell Signaling Technology).

**Fig. 1:**
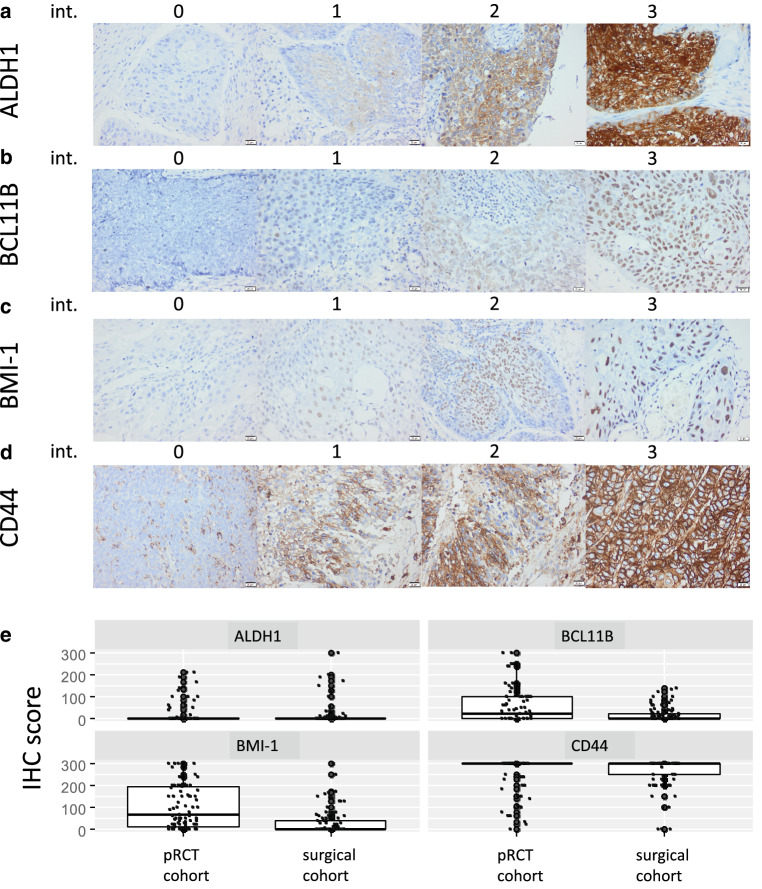
ALDH1, BCL11B, BMI‑1, and CD44 expression in head and neck squamous cell cancer (HNSCC) and distribution in the two study cohorts. Shown are examples of ALDH1 (**a**), BCL11B (**b**), BMI-1 (**c**), and CD44 (**d**) expression ranging from none (0), weak (1), and intermediate (2) to strong (3) in HNSCC (200 ×). ALDH1, BCL11B, BMI‑1, and CD44 staining is *red-brown*, while nuclei and cytoplasm are counter-stained with hemalaun (*blue*). **e** Immunohistochemistry (IHC) scores combine proportions and intensities of staining as described in “Material and methods” for the four reported stem cell markers ALDH1 (cytoplasmic expression), BCL11B (nuclear), BMI‑1 (nuclear), and CD44 (cytoplasmic). Per definition, IHC scores could reach values between 0 and 300. The plots reflect the frequency distribution of the IHC scores

In order to quantify and compare expression levels, immunohistochemistry score (IHC scores) were applied to all stainings. IHC scores represent the product of the percentages of positive cells and the staining intensity scored from negative (0), low (1), intermediate (2), to strong (3). They have a range of 0–300 (Fig. 1a–d).

### Clinical endpoints, survival analysis, and statistical analysis

Overall survival (OS), disease-free survival (DFS), and disease-specific survival (DSS) were chosen as clinical endpoints (in months). OS was calculated from the date of diagnosis to the point of death by any cause, DFS to the first observation of any recurrence or death, and DSS to the date of HNSCC-related death. In the absence of an event, patients were censored at the date of the last follow-up visit.

Survival data were analyzed by log rank test and visualized as Kaplan-Meier plots. To define dichotomous cut-off values for continuous IHC variables, an online tool (http://molpath.charite.de/cutoff/) was applied [23]. Univariate and multivariate analyses were conducted using Cox regression. A *p*-value < 0.05 was considered statistically significant. All further statistical procedures were conducted using SPSS, version 12.0 (IBM).

## Results

All 85 patients in the pRCT cohort were treated with primary radiotherapy or pRCT after diagnosis of advanced stage HNSCC. The 95 patients in the surgical cohort received surgical treatment with or without adjuvant radiotherapy. Detailed patient characteristics, risk factors, disease staging, treatment, and post-treatment follow-up including follow-up time of both cohorts are shown in Tables 1 and 2. Representative stainings and patterns of stem cell marker distributions are displayed in Fig. 1. The most abundant expression was detected for CD44, whereas it was absent for ALDH1 in most tumors. Deviations between the two study cohorts were most pronounced for BMI‑1 (*p* < 0.001, Mann-Whitney U test), less distinct for BCL11B (*p* = 0.016), and absent for ALDH1 (*p* = 0.922) and CD44 (*p* = 0.892). IHC staining was positively correlated in particular between BC11B and BMI‑1 in both study cohorts; however, the correlation coefficients did not exceed 0.5 in either case. Weaker positive correlations were observed between ALDH1 and BMI‑1 (pRCT cohort only) and between BMI‑1 and CD44 (both cohorts) (Table 3).

**Table 3:** Pairwise correlation coefficients as assessed by Kendall’s τ with respective *p*-values. p Values < 0.05 are highlighted in bold-italic. Italic = primary radio(chemo)therapy cohort, bold = surgical cohort

		ALDH1	BCL11B	BMI‑1	CD44
ALDH1	τ	–	*0.16*	*0.22*	*0.11*
	*p*	–	*0.119*	***0.015***	*0.266*
BCL11B	τ	**0.13**	–	*0.46*	*0.11*
	*p*	**0.162**	–	***<0.001***	*0.267*
BMI‑1	τ	**0.06**	**0.34**	–	*0.18*
	*p*	**0.534**	***<0.001***	–	***0.045***
CD44	τ	**0.18**	**0.17**	**0.24**	–
	*p*	**0.052**	**0.063**	***0.008***	–

### Baseline features and molecular markers in the pRCT cohort

**Fig. 2:**
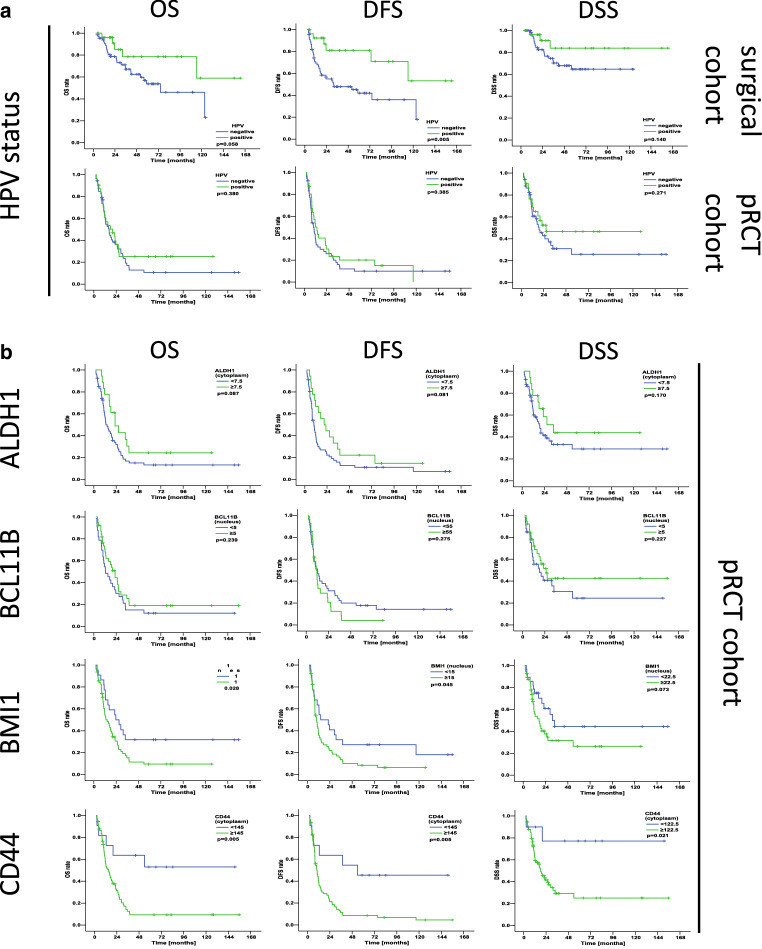
Association of human papillomavirus (HPV) status with clinical outcome in the surgical cohort and the primary radio(chemo)therapy (*pRCT*) cohort and association of single ALDH1, BCL11B, BMI‑1, and CD44 IHC scores with clinical outcome in the pRCT cohort. **a** Kaplan-Meier curves for the endpoints overall survival (*OS*), disease-free survival (*DFS*), and disease-specific survival (*DSS*) in head and neck squamous cell cancer (HNSCC) patients stratified according to HPV status (HPV-positive versus HPV-negative). *p*-Values (log-rank test) are indicated. **b** Kaplan-Meier curves for OS, DFS, and DSS in HNSCC patients stratified into groups with low and high expression of ALDH1, BCL11B, BMI‑1, and CD44. *p*-Values (log-rank test) and the applied optimized threshold for the classification of patient subgroups are indicated for the entire pRCT cohort

First, univariate Cox regression analysis was performed for baseline patient and tumor features with respect to OS, DFS, and DSS. Age was used as a continuous variable, while the other parameters are dichotomous (Supplemental Table 1). None of the baseline features exhibited a statistically significant relation with either OS, DFS, or DSS in the entire sample set (see Supplemental Table 1) or in the subsets stratified by HPV status (all *p* > 0.05). In particular, HPV status had no significant effect on survival (OS: *p* 0.38, DFS: *p* 0.39, DSS: *p* 0.27; log rank; Fig. 2a).

Thereafter, univariate Cox regression analysis was performed for the four investigated IHC parameters stained. For each staining parameter cut-off (CO), values were determined each for OS, DFS, and DSS. Analyses were each done for the entire pRCT cohort and, using the same COs, for the HPV-distinct subcohorts (Table 4). In the single marker analyses of the pRCT cohort, high expression of BMI‑1 was associated with a significantly decreased OS and DFS and a tendency to decreased DSS (OS: *p* 0.031, DFS: *p* 0.048, DSS: *p* 0.077; log rank). High expression of CD44 was associated with all types of survival (OS: *p* 0.008, DFS: *p* 0.007, DSS: *p* 0.036; log rank). High expression of ALDH1 showed a tendency to increased OS and DFS (OS: *p* 0.091, DFS: *p* 0.084; log rank). For the single marker analysis of BCL11B, no significant differences were observed (Table 4; Fig. 2b).

**Table 4:** Univariate Cox regression analysis for the four investigated immunohistochemistry parameters in the primary radio(chemo)therapy cohort. *P*-values < 0.05 are highlighted in bold. CO: cut-off values; HR (95% CI): hazard ratio with 95% confidence interval

	OS			DFS			DSS		
	CO	HR (95% CI)	*P*	CO	HR (95% CI)	*P*	CO	HR (95% CI)	*P*
*ALDH1*	7.5	0.59 (0.32–1.09)	0.091	7.5	0.61 (0.34–1.07)	0.084	7.5	0.60 (0.29–1.25)	0.175
*HPV−*	–	0.61 (0.28–1.33)	0.213	–	0.67 (0.32–1.40)	0.284	–	0.52 (0.20–1.37)	0.187
*HPV+*	–	0.49 (0.17–1.47)	0.204	–	0.47 (0.18–1.26)	0.135	–	0.65 (0.18–2.35)	0.512
*BCL11B*	5.0	0.73 (0.44–1.23)	0.241	55.0	1.33 (0.79–2.24)	0.277	5.0	0.68 (0.36–1.28)	0.231
*HPV−*	–	0.78 (0.40–1.50)	0.456	–	1.36 (0.69–2.68)	0.378	–	0.62 (0.28–1.39)	0.247
*HPV+*	–	0.67 (0.28–1.61)	0.369	–	1.16 (0.51–2.67)	0.724	–	0.79 (0.27–2.37)	0.679
*BMI‑1*	15.0	1.89 (1.06–3.37)	**0.031**	15.0	1.75 (1.01–3.03)	**0.048**	22.5	1.79 (0.94–3.42)	0.077
*HPV−*	–	1.89 (0.93–3.88)	0.081	–	1.85 (0.91–3.78)	0.090	–	1.66 (0.78–3.55)	0.192
*HPV+*	–	1.93 (0.71–5.27)	0.200	–	1.56 (0.62–3.92)	0.348	–	2.28 (0.63–8.22)	0.209
*CD44*	145.0	3.50 (1.39–8.83)	**0.008**	145.0	3.20 (1.37–7.47)	**0.007**	122.5	4.60 (1.11–19.11)	**0.036**
*HPV−*	–	2.34 (0.82–6.66)	0.110	–	2.36 (0.83–6.69)	0.107	–	4.52 (0.61–33.36)	0.139
*HPV+*	–	7.23 (0.96–54.65)	0.055	–	4.32 (0.99–18.78)	0.051	–	3.66 (0.47–28.56)	0.215

*OS* overall survival, *DFS* disease-free survival, *DSS* disease-specific survival

**Fig. 3:**
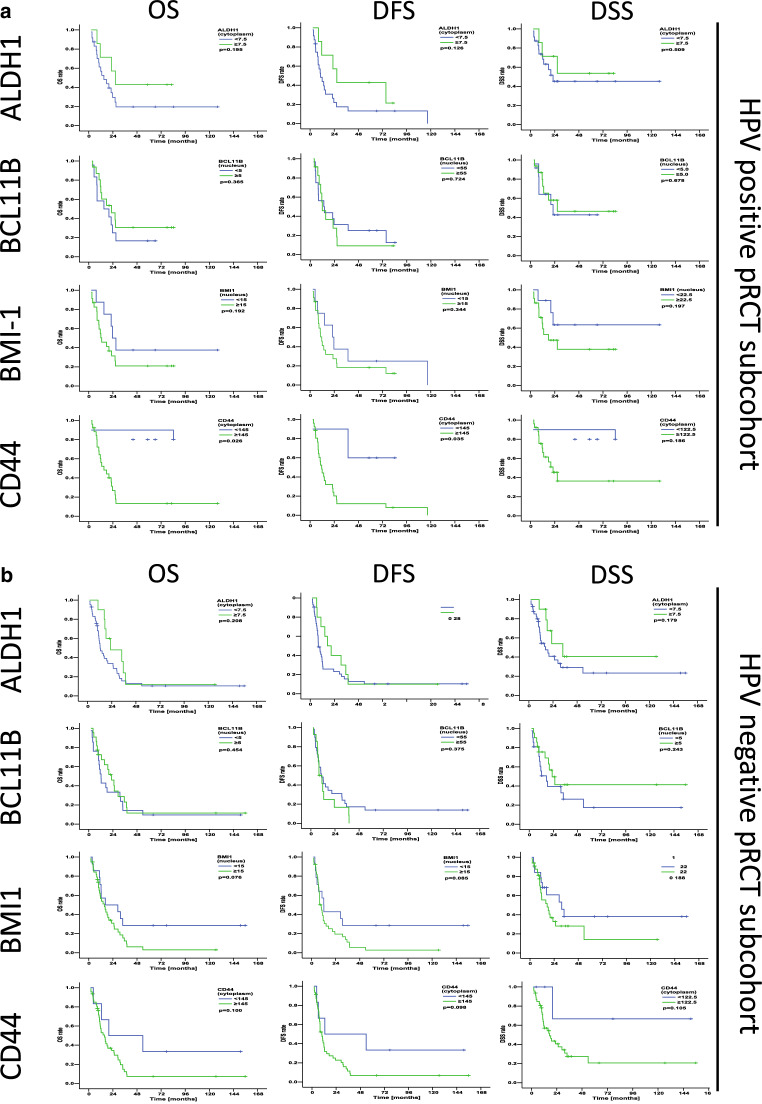
Association of single ALDH1, BCL11B, BMI‑1, and CD44 immunohistochemistry scores with clinical outcome in the human papillomavirus (*HPV*)-stratified primary radio(chemo)therapy (*pRCT*) subcohorts. Kaplan-Meier curves for overall survival (*OS*), disease-free survival (*DFS*), and disease-specific survival (*DSS*) in head and neck squamous cell carcinoma patients stratified into groups with low and high expression of ALDH1, BCL11B, BMI‑1, and CD44. *p*-Values (log-rank test) and the applied optimized threshold for the classification of patient subgroups are indicated for the HPV-positive subcohort (**a**) and the HPV-negative subcohort (**b**)

In the HPV-positive subcohort, no significant differences were observed between high and low marker expression. High expression of CD44 showed a tendency to decreased OS and DFS (OS: *p* 0.055, DFS: *p* 0.051; log rank; Table 4; Fig. 3a). In the HPV-negative subcohort, no significant differences were observed between high and low marker expression. High expression of BMI‑1 showed a tendency to decreased OS and DFS (OS: *p* 0.081, DFS: *p* 0.090; log rank; Table 4; Fig. 3b).

To assess covariate effects in the pRCT cohort, a multivariable Cox regression model comprising patient-, tumor-, and therapy-related features as well as the four IHC parameters (each stratified by the optimized threshold with regard to either OS, DFS, and DSS) was conducted. Of the patient-, tumor-, and therapy-related features, only concomitant chemotherapy was associated with favorable outcome (*p* 0.035, Table 5). In the HPV-stratified subcohorts, no significant associations were observed. For the four tested CSC markers, multivariable analysis showed significant prognostic influence of BMI‑1, with CD44 decreasing survival (BMI-1: OS, DFS, and DSS; CD44: OS, DFS and DSS), and ALDH1 and BCL11B supporting survival (ALDH1: OS, and DFS; BCL11B: OS, and DSS) (Table 5). In a multivariable analysis of the four tested CSC markers only, similar results were found for the whole pRCT cohort (Supplemental Table 2). For the HPV-negative subcohort, high expression of BMI‑1 was associated with significantly decreased OS. In the HPV-positive subcohort, OS was reduced with high expression of BMI‑1; DFS was decreased with high expression of CD44 and low expression of ALDH1 (Supplemental Table 2).

**Table 5:** Multivariable Cox regression model comprising patient-, tumor-, and therapy-related features as well as the four immunohistochemistry parameters in the primary radio(chemo)therapy (pRCT) cohort. *p-*Values < 0.05 are highlighted in bold. “Model” refers to the overall score encompassing all 13 variables. “Former HNSCC”: 14 patients were treated within the presented study for relapse of head and neck squamous cell cancer (HNSCC). “RT complete”: radiotherapy was completed in 82 patients and prematurely stopped in seven. “Chemotherapy”: 72 patients received chemotherapy concomitant to radiation

	OS			DFS			DSS		
Variable	Group	HR (95% CI)	*P*	Group	HR (95% CI)	*P*	Group	HR (95% CI)	*P*
*Age*	–	0.99 (0.96–1.02)	0.352	–	1.00 (0.97–1.03)	0.872	–	1.00 (0.97–1.04)	0.926
*Gender*	Female vs male	1.51 (0.67–3.39)	0.324	–	0.81 (0.38–1.71)	0.581	–	0.57 (0.20–1.58)	0.276
*T stage*	cT4 vs cT1–cT3	0.70 (0.31–1.59)	0.390	–	0.90 (0.43–1.85)	0.764	–	1.27 (0.41–3.90)	0.678
*N stage*	cN+ vs cN0	0.70 (0.29–1.72)	0.439	–	0.56 (0.25–1.26)	0.162	–	0.94 (0.27–3.25)	0.921
*Grading*	G3 vs G1–G2	1.90 (0.85–4.25)	0.116	–	1.46 (0.66–3.22)	0.346	–	1.70 (0.69–4.23)	0.252
*HPV*	HPV+ vs HPV−	1.34 (0.68–2.65)	0.401	–	0.88 (0.46–1.68)	0.694	–	1.02 (0.45–2.33)	0.965
*Former HNSCC*	Yes vs no	0.77 (0.32–1.88)	0.571	–	1.09 (0.47–2.51)	0.839	–	1.10 (0.30–3.97)	0.886
*RT complete*	Yes vs no	0.76 (0.22–2.61)	0.666	–	0.58 (0.19–1.80)	0.341	–	0.48 (0.13–1.72)	0.259
*Chemotherapy*	Yes vs no	0.44 (0.20–0.94)	**0.035**	–	0.58 (0.28–1.20)	0.140	–	0.58 (0.24–1.40)	0.226
*ALDH1*	≥7.5 vs <7.5	0.39 (0.20–0.79)	**0.009**	≥7.5 vs <7.5	0.34 (0.17–0.69)	**0.003**	≥7.5 vs <7.5	0.45 (0.20–1.05)	0.064
*BCL11B*	≥5.0 vs <5.0	0.31 (0.15–0.64)	**0.002**	≥55.0 vs 7.5	0.63 (0.32–1.28)	0.201	≥5.0 vs <5	0.41 (0.18–0.96)	**0.039**
*BMI‑1*	≥15.0 vs <15.0	3.86 (1.69–8.82)	**0.001**	≥15.0 vs <15.0	2.43 (1.13–5.19)	**0.022**	≥22.5 vs <22.5	3.30 (1.15–9.47)	**0.027**
*CD44*	≥145.0 vs <145.0	8.23 (2.30–29.5)	**0.001**	≥145.0 vs <145.0	4.87 (1.62–14.6)	**0.005**	122.5	9.20 (1.10–76.7)	**0.040**
*Model*	–	χ^2^ = 35.89	**0.001**	–	χ^2^ = 29.47	**0.006**	–	χ^2^ = 22.17	0.053

*OS* overall survival, *DFS* disease-free survival, *DSS* disease-specific survival

### Baseline features and molecular markers in the surgical cohort

Accordingly, univariate Cox regression analysis was performed for baseline patient and tumor features with respect to OS, DFS, and DSS, in the surgical cohort. Age was used as a continuous variable, while the other parameters are dichotomous (Table 6).

**Table 6:** Univariate Cox regression analysis for baseline patient and tumor features in the surgical cohort. *p*-Values < 0.05 are highlighted in bold. HR (95% CI): hazard ratio with 95% confidence interval

	OS		DFS		DSS	
	HR (95% CI)	*P*	HR (95% CI)	*P*	HR (95% CI)	*P*
Age (per year)	1.04 (1.00–1.07)	**0.035**	1.03 (1.00–1.06)	0.054	1.04 (1.00–1.08)	0.054
*HPV−*	1.03 (0.99–1.07)	0.126	1.03 (0.99–1.07)	0.112	1.03 (0.98–1.07)	0.224
*HPV+*	1.12 (0.99–1.27)	0.064	1.10 (0.98–1.23)	0.119	1.12 (0.96–1.29)	0.144
Female vs male	1.00 (0.44–2.25)	0.997	1.01 (0.50–2.07)	0.973	1.28 (0.51–3.23)	0.601
*HPV−*	1.13 (0.47–2.72)	0.786	1.21 (0.57–2.59)	0.617	1.50 (0.56–4.00)	0.422
*HPV+*	<5 events	–	<5 events	–	<5 events	–
pT4 vs pT1–3	1.54 (0.74–3.24)	0.251	1.18 (0.62–2.24)	0.611	1.58 (0.65–3.81)	0.312
*HPV−*	1.33 (0.57–3.09)	0.514	1.01 (0.50–2.06)	0.979	1.48 (0.55–3.95)	0.435
*HPV+*	3.63 (0.36–36.57)	0.275	2.00 (0.22–18.49)	0.542	5.02 (0.43–58.91)	0.199
pN+ vs pN0	1.28 (0.57–2.84)	0.550	0.97 (0.51–1.84)	0.918	1.55 (0.58–4.17)	0.387
*HPV−*	3.05 (1.04–8.93)	**0.042**	1.57 (0.74–3.35)	0.245	4.68 (1.07–20.48)	**0.040**
*HPV+*	0.47 (0.05–4.55)	0.515	0.78 (0.09–7.08)	0.822	0.32 (0.03–3.59)	0.357
G3 vs G1/2	0.48 (0.17–1.36)	0.165	0.47 (0.20–1.10)	0.083	0.53 (0.16–1.77)	0.301
*HPV−*	1.03 (0.31–3.47)	0.960	1.01 (0.39–2.60)	0.991	1.41 (0.41–4.90)	0.585
*HPV+*	0.35 (0.04–3.20)	0.355	0.28 (0.03–2.45)	0.252	<5 events	–
R1 vs R0	6.34 (2.42–16.63)	**0.0002**	3.68 (1.44–9.38)	**0.006**	5.94 (1.76–20.10)	**0.004**
*HPV−*	4.30 (1.45–12.77)	0.009	2.52 (0.88–7.19)	0.084	3.46 (0.79–15.15)	0.100
*HPV+*	<5 events	–	<5 events	–	<5 events	–
HPV+ vs HPV−	0.40 (0.15–1.06)	0.066	0.31 (0.13–0.73)	**0.008**	0.41 (0.12–1.39)	0.153

In the surgical cohort, the authors found a significant reduction of survival in patients with R1-resection (OS: *p* 0.0002, DFS: *p* 0.006, DSS: *p* 0.004; log rank). Also, DFS was significantly decreased in patients with no HPV association; OS only tended to decrease (DFS: *p* 0.008, OS: *p* 0.066; log rank; Table 6; Fig. 2a). In the HPV-negative subcohort, node metastasis significantly decreased both OS and DSS (OS: *p* 0.042, DSS: *p* 0.040; log rank; Table 6).

Thereafter, univariate Cox regression analysis was performed for the four investigated IHC parameters stained, accordingly to the pRCT cohort. For each staining parameter, CO values were determined each for OS, DFS, and DSS, using the same COs for all (sub)cohorts. None of the four investigated IHC parameters exhibited a statistically significant relation with either OS, DFS, or DSS in the entire sample set (Supplemental Table 3) or in the subsets stratified by HPV status (all *p* > 0.05). Therefore, no multivariable Cox regression model was conducted in the surgical cohort.

## Discussion

In HNSCC, classical prognosticators include age, comorbidities, performance status, frailty, and advanced tumor stage [24], as well as nodal status [25], especially extracapsular extension [26], and close or involved margin resection [26, 27]. Especially in advanced stage cancer, data and knowledge on prognosis are very limited [28]. For some time, HPV association has been a known factor in HNSCC prognosis [29, 30]. Therefore, the International Collaboration on Oropharyngeal cancer Network for Staging (ICON-S) proposed an alternative staging system that eventually led to the independent HPV-positive oropharyngeal cancer classification in the 8th edition of the Union for International Cancer Control/American Joint Committee on Cancer TNM classification [8]. The data presented here support this hypothesis, but only in the surgical cohort.

While in the surgical cohort R status, HPV status, and nodal status were prognostic survival markers (worse prognosis after R1 resection, in HPV−, and HPV− N+ disease), these factors, and especially HPV status, were not relevant factors for prognosis in the pRCT cohort. In early stage cancer, prognosis may be driven by cancer biology such as tumor stage, histological grade, and mutation status, whereas prognostic variables in patients with advanced cancer tend to consist of patient-related factors such as performance status and cancer anorexia/cachexia [31]. In the current study, the prognostic indifference of HPV status in the pRCT cohort might be explained by a selection bias of relatively sick patients with a high number of relevant comorbidities and low performance status. Unfortunately, robust data on performance status was not available in most of the older cases.

Interestingly, the authors did not see any significant effect of the tested CSC markers in the surgical cohort. A possible explanation is too short a follow-up time. One other reason might be a rather small sample size being responsible for no significance in the univariate analyses of the tested molecular markers, especially in the surgical cohort. In general, there might be a relevant selection bias in the surgical cohort, since many primarily identified patients needed to be excluded as described in “Material and methods”. Regardless, the molecular CSC markers had a significant influence in the pRCT cohort with a comparable sample size and an only slightly longer follow-up time. Although TNM staging seems similar in both cohorts, the two cohorts cannot be considered fully comparable since co-morbidities and especially non-resectability might be strong confounders for survival. In this retrospective investigation, it was inconclusive in some cases what exactly triggered the therapy decision, especially in the pRCT cohort. Presumably, overall prognosis in the pRCT cohort is a priori worse than in the surgical cohort. Therefore, results of the cohorts must be seen independently from each other. In general, the retrospective manner of the investigation and missing validation in independent cohorts are limitations of the study design.

Recently, the number of molecular markers linked to survival increased considerably [26]. The identification of new markers in cancer is crucial to develop better forecasts on prognosis and to find new therapy options [5, 7, 19, 32]. In this field of research, CSC markers are very prominent, although some are linked to a better and some to a worse prognosis, whereas the literature is controversial for some markers [12, 13, 33, 34].

Of the CSC markers, CD44 is of the highest clinical relevance. It was demonstrated that expression of CD44 isoforms in HNSCC is differently associated with advanced T stage, regional and distant metastasis, and radiation failure, which suggests an involvement of CD44 in HNSCC tumor cell proliferation and migration [35]. In the present surgical cohort, different CSC marker expression did not alter survival prognosis, but in the pRCT cohort, high CD44 expression was associated with poorer survival (OS, DFS, and DSS). These results support the findings that identified high levels of CD44 mRNA, CD44 protein, and SCL3A2 mRNA expression as prognosticators for local recurrence in HNSCC after adjuvant RCT [36]. These findings have been validated for an independent patient cohort with locally advanced HNSCC after adjuvant RCT and for a cohort of HNSCC patients receiving pRCT [36, 37]. CD44 levels have also been correlated with clinical response to radiotherapy and may predict local recurrence in patients with early-stage laryngeal cancers and local recurrence and progression-free survival in oropharyngeal SCC [38, 39]. While CD44 plays an emerging role in HNSCC prognostication, the development of an anti-CD44 immunoconjugate bivatuzumab mertansine for therapeutic use in patients with advanced HNSCC was terminated after the immunoconjugate resulted in skin toxicity [40]. In oropharyngeal SCC patients, low CD44 expression levels in combination with HPV positivity were found to be positively associated with 3‑year DFS and OS after undergoing different therapy regimens [41].

The polycomb complex protein BMI‑1 is frequently overexpressed in HNSCC and increased BMI‑1 expression was associated with cervical node metastasis, Ki-67 abundance, and reduced OS and also served as an independent prognostic factor for patient outcomes in oral cavity SCC [42]. While some studies could not predict survival from BMI‑1 expression [43, 44], other studies demonstrated decreased radiosensitivity and increased probability for distant metastases in an experimental setting [45]. The data presented here demonstrate different perspectives on BMI‑1 in HNSCC: While it was not associated with survival in the surgical cohort, BMI‑1 was a strong and negative prognosticator in the pRCT cohort. Frequently, BMI‑1 has been linked to other CSC markers such as ALDH1 and BCL11B [20, 45, 46].

ALDH family proteins belong to the most studied CSC markers in HNSCC. These proteins are increased under cisplatin treatment and radiotherapy and mediate CSC survival in HNSCC [14]. Targeting of ALDH+ cells decreased tumor burden and sensitized HNSCC cells for cisplatin treatment [47]. ALDH1 was well correlated with tumor size, lymph node metastasis, and histopathological grading in HNSCC, being predominantly found in more aggressive tumors and higher tumor stage [14, 17, 48]. ALDH1 and other proteins from the ALDH family belong to the most prevalent markers to identify CSC in HNSCC [34]. ALDH1 expression was linked to treatment resistance, CSC-like properties, higher circulating myeloid-derived suppressor cells, and poor prognosis as well as angiolympathic invasion in oral cavity SCC [49, 50]. Although in the literature high ALDH1 expression was mostly correlated to worse prognosis [51, 52], the authors could not show an effect on survival in their surgical cohort. In the pRCT cohort, high ALDH1 even correlated with better outcome in the multivariate analysis and tended to do so in univariate analysis.

Strongly linked to BMI‑1, BCL11B was suggested as a marker to identify CSCs in HNSCC [20]. This link is supported by the authorsʼ findings in the correlation analysis of the tested CSC markers. Predominantly studied in T‑cell malignancies, high BCL11B expression indicated a favorable outcome in patients [20, 53]. Similarly, the authors could show in their data that high expression of BCL11B was protective in the multivariate analysis of the CSC markers in the pRCT cohort.

Although all of the markers are discussed as identifying CSCs, it is possible that the authorsʼ data show a differential perspective on the four tested markers regarding prognosis in the pRCT cohort. One explanation might be a heterogeneity in CSCs, with different types or states of CSCs promoting disease progression and others slowing it down. The present data suggest that CD44 and BMI‑1 are linked to poorer prognosis, whereas ALDH1 and BCL11B are linked to tendentially better prognosis.

All studies on clinical correlations of the expression of CSC markers, including the present study, have only limited scope to consider intratumoral heterogeneity as well as tumor/metastasis heterogeneity. Experimental data suggest that expression profiles of CSC and EMT markers may vary within tumors and during metastasizing [54]. Innovative approaches are warranted in order to take into account these aspects in the future. In the age of precision medicine and increasing numbers of approaches in HNSCC treatment, CSC markers, such as ALDH, CD44, BMI‑1, and Sox2, as well as c‑Met and EGFR, yield opportunities to further stratify prognosticators and gain therapy targets [34, 55]. The authorsʼ data emphasize the role of interdisciplinary clinical conferences especially in advanced stage patients with comorbidities to thoroughly identify separate prognostic features and search for alternate targeted therapy approaches.

## Conclusion

This two cohort study is the first to show remarkable differences for prognosticators in HNSCC between patients that received either surgical treatment, with or without adjuvant therapy, or were treated with pRCT .

In the pRCT cohort of advanced stage HNSCC patients, CSC markers had a differential effect on survival, while HPV status had no significant influence. Of the CSC markers, BMI‑1 and CD44 indicated a significantly worse prognosis on survival. However, ALDH1 and BCL11B appear to have protective properties in HNSCC survival.

In a cohort with mostly advanced stage HNSCC patients that were suitable for and treated with surgery and with or without adjuvant radio(chemo)therapy, classical prognostic factors such as HPV status, R1 resection, and nodal status in HPV-negative HNSCC played a significant role in survival prognosis, but the tested CSC markers showed no significant effect on survival prognosis.

In conclusion, testing for CSC markers might be beneficial in patients with advanced or late stage HNSCC, especially if many comorbidities exist or disease is inoperable. In these cases, and if the findings can be validated in independent cohorts, early molecular testing in interdisciplinary clinical conferences might be beneficial, e.g., for earlier application of targeted therapies.

## Caption Electronic Supplementary Material

Supplementary Table 1: Univariate Cox regression analysis for baseline patient and tumor features in the pRCT cohort. Supplementary Table 2: Multivariable Cox regression model with the four IHC parameters in the pRCT cohort. Supplementary Table 3: Univariate Cox regression analysis for the four investigated IHC parameters in the surgical cohort.
