# Supplementary material for: Role of cancer stem cell markers ALDH1, BCL11B, BMI-1, and CD44 in the prognosis of advanced HNSCC
Source: Strahlenther Onkol. 2020 Jun 25;197(3):231–45. doi: 10.1007/s00066-020-01653-5 (PMC7892527; doi:10.1007/s00066-020-01653-5)
Supplement: Supplementary file 1 — Supplementary Table 1: Univariate Cox regression analysis for baseline patient and tumor features in the pRCT cohort. Supplementary Table 2: Multivariable Cox regression model with the four IHC parameters in the pRCT cohort. Supplementary Table 3: Univariate Cox regression analysis for the four investigated IHC parameters in the surgical cohort. [file 66_2020_1653_MOESM1_ESM.docx]

Supplemental Table 1: Univariate Cox regression analysis for baseline patient and tumor features in the pRCT cohort. HR (95% CI): hazard ratio with 95% confidence interval.

|  | OS | | DFS | | DSS | |
| --- | --- | --- | --- | --- | --- | --- |
|  | HR (95% CI) | P | HR (95% CI) | P | HR (95% CI) | P |
| Age (per year) | 1.00  (0.98-1.02) | 0.711 | 1.00  (0.97-1.02) | 0.632 | 0.99  (0.97-1.02) | 0.519 |
| HPV- | 1.02  (0.99-1.05) | 0.291 | 1.02  (0.99-1.05) | 0.309 | 1.00  (0.96-1.03) | 0.828 |
| HPV+ | 0.98  (0.94-1.02) | 0.342 | 0.98  (0.94-1.02) | 0.228 | 1.00  (0.95-1.05) | 0.947 |
| Female vs male | 0.88  (0.46-1.67) | 0.687 | 0.89  (0.49-1.62) | 0.713 | 0.92  (0.43-1.98) | 0.838 |
| HPV- | 0.71  (0.32-1.60) | 0.407 | 0.77  (0.36-1.66) | 0.504 | 0.73  (0.28-1.90) | 0.517 |
| HPV+ | 2.04  (0.66-6.31) | 0.215 | 1.46  (0.48-4.42) | 0.501 | 2.02  (0.55-7.42) | 0.290 |
| cT4 vs cT1-3 | 0.86  (0.50-1.49) | 0.598 | 0.92  (0.55-1.54) | 0.744 | 1.43  (0.67-3.06) | 0.356 |
| HPV- | 1.09  (0.50-2.34) | 0.836 | 1.07  (0.51-2.22) | 0.865 | 1.20  (0.46-3.13) | 0.711 |
| HPV+ | 0.64  (0.26-1.59) | 0.340 | 0.60  (0.25-1.41) | 0.237 | 1.95  (0.43-8.75) | 0.385 |
| cN+ vs cN0 | 0.85  (0.45-1.61) | 0.614 | 0.77  (0.42-1.40) | 0.387 | 1.13  (0.48-2.66) | 0.780 |
| HPV- | 0.98  (0.38-2.49) | 0.959 | 0.95  (0.37-2.42) | 0.910 | 1.14  (0.35-3.76) | 0.832 |
| HPV+ | 0.74  (0.27-2.00) | 0.546 | 0.49  (0.20-1.19) | 0.113 | 1.32  (0.29-5.93) | 0.717 |
| G3 vs G1/2 | 1.45  (0.82-2.57) | 0.204 | 1.19  (0.68-2.10) | 0.543 | 1.62  (0.82-3.18) | 0.163 |
| HPV- | 1.54  (0.76-3.14) | 0.230 | 1.36  (0.67-2.74) | 0.398 | 1.88  (0.84-4.20) | 0.127 |
| HPV+ | 1.34  (0.49-3.63) | 0.572 | 0.99  (0.37-2.63) | 0.977 | 1.29  (0.36-4.65) | 0.696 |
| HPV+ vs HPV- | 0.80  (0.48-1.33) | 0.38 | 0.81  (0.50-1.31) | 0.39 | 0.70  (0.37-1.32) | 0.27 |

Supplemental Table 2: Multivariable Cox regression model with the four IHC parameters in the pRCT cohort. P values < 0.05 are highlighted in bold.

|  | OS | | | DFS | | | DSS | | |
| --- | --- | --- | --- | --- | --- | --- | --- | --- | --- |
|  | CO | HR (95% CI) | P | CO | HR (95% CI) | P | CO | HR (95% CI) | P |
| HPVall |  |  |  |  |  |  |  |  |  |
| ALDH1 | 7.5 | 0.42  (0.22-0.83) | **0.012** | 7.5 | 0.37  (0.19-0.70) | **0.002** | 7.5 | 0.50  (0.23-1.10) | 0.085 |
| BCL11B | 5.0 | 0.51  (0.29-0.91) | **0.023** | 55.0 | 0.85  (0.48-1.51) | 0.585 | 5.0 | 0.47  (0.23-0.97) | **0.040** |
| BMI-1 | 15.0 | 3.10  (1.47-6.53) | **0.003** | 15.0 | 2.39  (1.15-4.97) | **0.019** | 22.5 | 3.21  (1.34-7.73) | **0.009** |
| CD44 | 145.0 | 4.80  (1.67-13.82) | **0.004** | 145.0 | 3.95  (1.50-10.4) | **0.005** | 122.5 | 7.06  (0.96-52.2) | 0.056 |
| Model |  | χ²=25.07 | **5*10^-5^** |  | χ²=19.47 | **0.001** |  | χ²=16.81 | **0.002** |
|  |  |  |  |  |  |  |  |  |  |
| HPV- |  |  |  |  |  |  |  |  |  |
| ALDH1 | 7.5 | 0.46  (0.20-1.11) | 0.083 | 7.5 | 0.45  (0.19-1.05) | 0.063 | 7.5 | 0.50  (0.18-1.38) | 0.178 |
| BCL11B | 5.0 | 0.57  (0.28-1.18) | 0.131 | 55.0 | 0.87  (0.41-1.82) | 0.710 | 5.0 | 0.51  (0.22-1.23) | 0.135 |
| BMI-1 | 15.0 | 3.68  (1.34-10.13) | **0.012** | 15.0 | 3.28  (1.17-9.23) | **0.024** | 22.5 | 2.89  (0.99-8.45) | 0.053 |
| CD44 | 145.0 | 2.36  (0.77-7.25) | 0.134 | 145.0 | 1.96  (0.65-5.87) | 0.232 | 122.5 | 2.28  (0.29-17.71) | 0.430 |
| Model |  | χ²=11.29 | **0.024** |  | χ²=8.33 | 0.080 |  | χ²=8.01 | 0.091 |
|  |  |  |  |  |  |  |  |  |  |
| HPV+ |  |  |  |  |  |  |  |  |  |
| ALDH1 | 7.5 | 0.30  (0.08-1.13) | 0.075 | 7.5 | 0.16  (0.04-0.63) | **0.009** | 7.5 | 0.38  (0.08-1.68) | 0.200 |
| BCL11B | 5.0 | 0.38  (0.11-1.24) | 0.108 | 55.0 | 0.60  (0.22-1.61) | 0.307 | 5.0 | 0.20  (0.04-1.04) | 0.056 |
| BMI-1 | 15.0 | 4.16  (1.05-16.51) | **0.042** | 15.0 | 1.80  (0.55-5.86) | 0.332 | 22.5 | 9.74  (1.37-69.50) | **0.023** |
| CD44 | 145.0 | n.a. | - | 145.0 | 27.0  (2.84-256.4) | **0.004** | 122.5 | n.a. | - |
| Model |  | χ²=16.15 | **0.003** |  | χ²=15.89 | **0.003** |  | χ²=9.43 | 0.051 |

Supplemental Table 3: Univariate Cox regression analysis for the four investigated IHC parameters in the surgical cohort.

|  | OS | | | DFS | | | DSS | | |
| --- | --- | --- | --- | --- | --- | --- | --- | --- | --- |
|  | CO | HR (95% CI) | P | CO | HR (95% CI) | P | CO | HR (95% CI) | P |
| ALDH1 | 25 | 0.65  (0.19-2.25) | 0.499 | 3.75 | 0.63  (0.28-1.43) | 0.267 | 1.25 | 0.60  (0.18-2.05) | 0.416 |
| HPV- |  | 1.54  (0.32-7.50) | 0.596 |  | 0.81  (0.31-2.12) | 0.673 |  | 0.73  (0.17-3.18) | 0.675 |
| HPV+ |  | 0.52  (0.06-4.79) | 0.561 |  | 0.80  (0.15-4.41) | 0.797 |  | 0.80  (0.07-8.77) | 0.852 |
| BCL11B | 17.5 | 1.57  (0.75-3.28) | 0.230 | 51.25 | 1.60  (0.74-3.48) | 0.233 | 51.25 | 2.31  (0.85-6.32) | 0.103 |
| HPV- |  | 1.25  (0.56-2.75) | 0.588 |  | 1.15  (0.50-2.65) | 0.748 |  | 1.51  (0.50-4.60) | 0.468 |
| HPV+ |  | 2.08  (0.21-20.30) | 0.530 |  | < 5 events | - |  | < 5 events | - |
| BMI-1 | 62.5 | 0.83  (0.34-2.06) | 0.692 | 1.25 | 1.51  (0.82-2.78) | 0.187 | 42.5 | 0.63  (0.21-1.87) | 0.403 |
| HPV- |  | 0.65  (0.24-1.74) | 0.388 |  | 1.12  (0.58-2.17) | 0.741 |  | 0.55  (0.18-1.68) | 0.293 |
| HPV+ |  | < 5 events | - |  | 1.95  (0.36-10.75) | 0.442 |  | < 5 events | - |
| CD44 | 212.5 | 1.18  (0.48-2.90) | 0.717 | 266.7 | 1.24  (0.61-2.54) | 0.550 | 291.7 | 0.84  (0.34-2.09) | 0.712 |
| HPV- |  | 0.71  (0.26-1.90) | 0.492 |  | 1.15  (0.50-2.63) | 0.749 |  | 0.67  (0.25-1.79) | 0.428 |
| HPV+ |  | 3.17  (0.33-30.36) | 0.316 |  | 0.89  (0.17-4.61) | 0.893 |  | 1.31  (0.12-14.52) | 0.824 |
